# Adverse events of antibody–drug conjugates on the ocular surface in cancer therapy

**DOI:** 10.1007/s12094-023-03261-y

**Published:** 2023-07-15

**Authors:** Sandra Domínguez-Llamas, Manuel Caro-Magdaleno, Beatriz Mataix-Albert, Javier Avilés-Prieto, Isabel Romero-Barranca, Enrique Rodríguez-de-la-Rúa

**Affiliations:** 1 Department of Ophthalmology, University Hospital Virgen Macarena, Políclínico 2a planta, Avda. Dr. Fedriani 3, 41009 Seville, Spain; 2 Department of Surgery, Ophthalmology Area, University of Seville, Seville, Spain

**Keywords:** Antibody–drug conjugates, Ocular surface, Adverse events, Ocular toxicity, Cancer therapy

## Abstract

Antibody–drug conjugates consist of a monoclonal antibody attached to a cytotoxic therapeutic molecule by a connector. This association allows a highly specific therapy, which increases their effectiveness and decreases their potential toxicity. This new therapy emerged approximately 20 years ago; since then, numerous combinations have appeared in the field of treatment-related neoplasms as an alternative for patients who do not achieve good results with conventional treatment options. Adverse effects of these drugs on the ocular surface are frequent and varied. Their prevalence ranges from 20 to 90% depending on the drug and administration condition, probably due to multiple receptor-mediated factors or mechanisms not mediated by specific receptors, such as macropinocytosis. These adverse events can greatly limit patients’ comfort; thus, the objectives of this article were, in the first place, to compile the information currently available on different types of adverse effects of antibody–drug conjugates on the ocular surface, including pathophysiology, prevalence, and treatment, and in second place, to contribute to the correct identification and management of these events, which will result in a lower rate of cessation of treatment, which is necessary for the survival of candidate patients.

## What are antibody–drug conjugates (ADCs)?

**Fig. 1:**
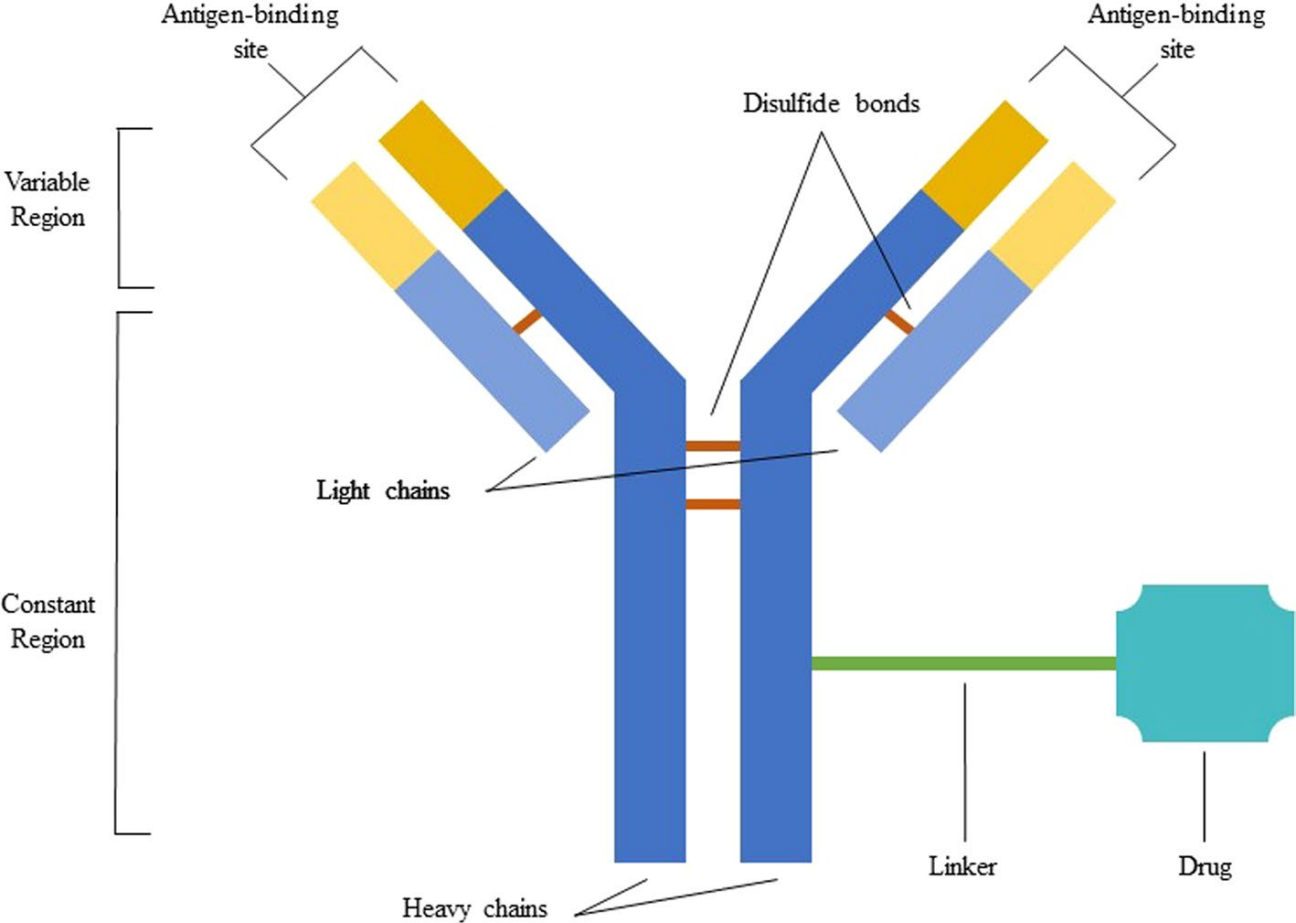
ADC structure. *ADC* antibody–drug conjugate

ADCs form by the binding of a cytotoxic molecule to a monoclonal antibody via a chemical linker, as depicted in Fig. 1. ADCs are useful as the binding allows monoclonal antibodies to transport molecules with therapeutic capacity specifically to target cells. These cells internalize the complex by endocytosis or pinocytosis, disrupt the union with the help of their lysosomes, and release the cytotoxic molecule, possibly causing their death through apoptosis or other mechanisms [1–4].

The binding specificity reduces the systemic exposure of the drug to a minimum, thereby limiting its action in healthy tissues and, consequently, the undesirable side effects. Thus, the use of ADCs can increase the effectiveness of drugs in the treatment of hematological cancers and cancers of solid organs by reducing therapeutic limitations due to drug intolerance [1, 3].

## Proposed pathophysiology of adverse effects (AEs) on the ocular surface caused by ADCs

The basic advantage of ADCs is that their action is specifically directed at target cells through the selection of monoclonal antibodies against specific targets. This process has markedly decreased toxicity compared with other conventional chemotherapies, although the side effects cannot be completely eradicated.

The information obtained from the clinical use of different ADCs that are currently available indicates that the eye is one of the most frequently affected organs by drug-related Aes. There are different reasons why ADCs generate Aes on the ocular surface, such as the existence of a large blood flow, fast-growing cell subpopulations, and an abundant variety of cell surface receptors. Moreover, their toxicity can be mediated by multiple mechanisms, which are classified into two types: off-target toxicity and target toxicity [2].

Numerous off-target mechanisms have been described. The cytotoxic molecule of the ADC may be released early because of unstable binding with its monoclonal antibody due to an inadequate linker [2]. The cytotoxic molecule may exert its action in the wrong territory, thereby causing damage. Moreover, it also possible that the damage is caused by intracellular metabolism by which the linker is separated from the cytotoxin. In this process, ionized intermediate metabolites do not diffuse through the cell membrane and accumulate in the cytoplasm, resulting in cell damage [5]. Free payloads can enter the extracellular space by passive diffusion due to the permeability of the cell membrane or high lipophilicity of the payloads or be released because of the loss of cell integrity after cell death. Subsequently, these free cytotoxic molecules can access the intracellular space of another cell through off-target mediated mechanisms such as passive diffusion, transporter-mediated uptake, or non-specific endocytosis mechanisms to cause cell damage, referred to as the bystander effect [6].

Endocytosis is another important off-target means of entry into healthy cells. This mechanism can be divided into pinocytosis, a receptor-independent and non-specific way of internalizing extracellular fluid and solutes, and phagocytosis, a receptor-dependent and non-specific internalization of larger opsonized particulate matter. Endocytosis can be classified into macroendocytosis (0.2–10 μM) and microendocytosis (< 200 nm), depending on the size of the endocytic vacuoles. Among the different mechanisms of non-specific endocytosis, the most important pathway is macropinocytosis [6, 7]. Some ADCs reach the ocular surface through the tear film or perilimbal vessels. For example, the presence of belamaf in tears at a non-quantifiable dose was demonstrated in a study conducted on rabbits receiving 15–30 mg/kg/week of belantamab mafodotin for 1 month [8]. ADCs are subsequently internalized via macropinocytosis, which leads to cell apoptosis [5, 8, 9]. The chemical structure of the ADC molecule influences the capacity for cellular internalization as macromolecules, with the presence of more positive charges or hydrophobic residues on the surface favoring macropinocytosis. Therefore, these chemical characteristics increase off-target toxicity in the corneal epithelium [9]. It is postulated that certain non-specific receptors, such as Fc gamma receptors (FcγRs), neonatal Fc receptor, and C-type lectin receptors, can mediate ADC phagocytosis by interacting with the Fc region of the monoclonal antibody present in the molecule [6]. Due to the high level of corneal turnover, the apoptotic cells advance toward the corneal center, as indicated by Thoft’s hypothesis of cell migration from the limbus to the corneal center. The existence of microcysts at the corneal level causes a decrease in visual acuity. At the level of the corneal periphery, they produce oblate-type topographic alterations simulating post-myopic surgery topography, and at the central level, the loss of media transparency is observed [10]. Furthermore, keratitis and ulcers can be produced by epithelial alterations that cause great discomfort to the patient, forcing temporary discontinuation of the drug and sometimes total suspension [8, 11].

**Fig. 2:**
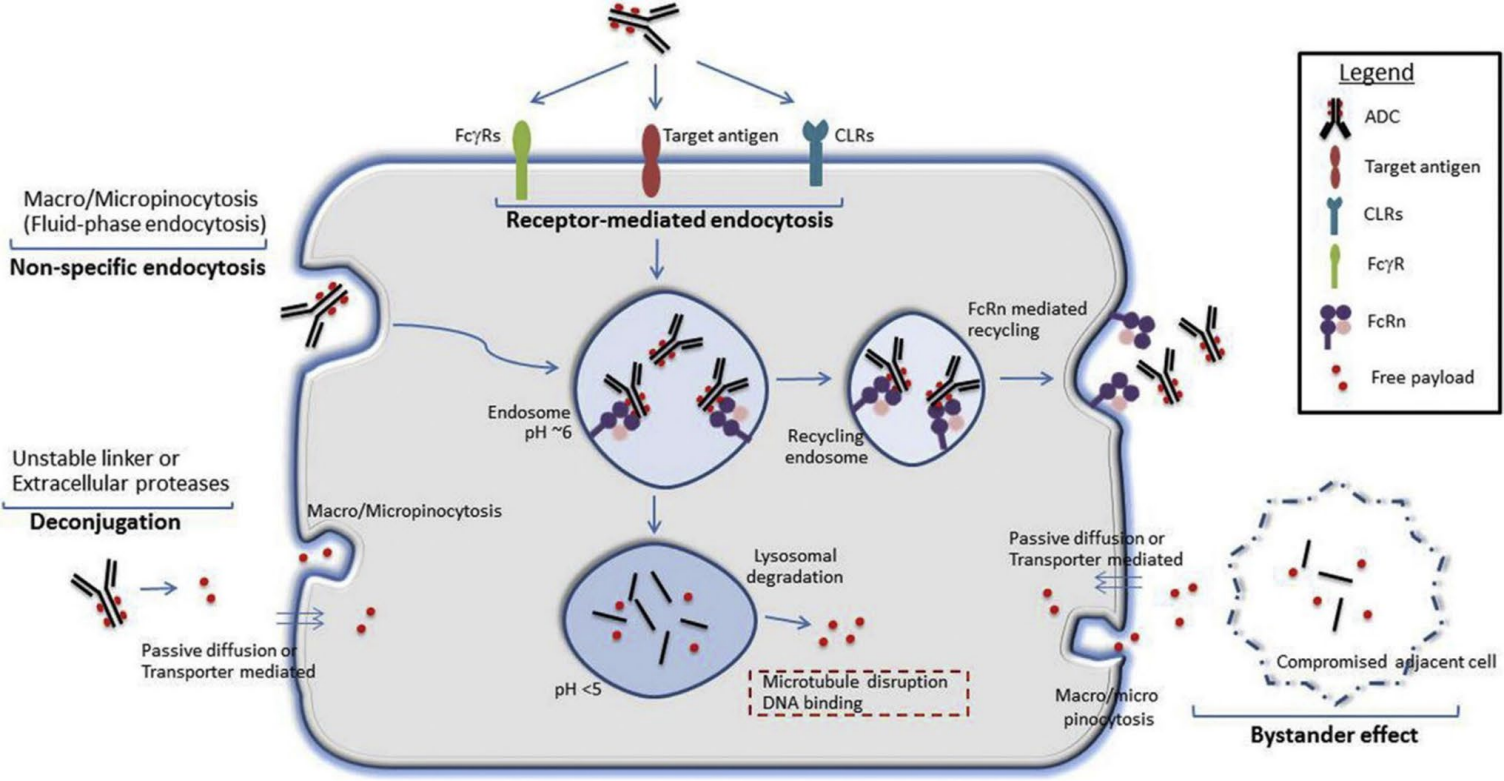
Possible mechanisms of access to the cellular interior of ADCs. Reprinted from Pharmacology & Therapeutics, 200, Mahalingaiah PK, Ciurlionis R, Durbin KR, et al. Potential mechanisms of target-independent uptake and toxicity of antibody–drug conjugates, p113 (2019), with permission from Elsevier. [25]. *ADC* antibody–drug conjugate. *FcγRs* Fc gamma receptors, *FcRn* neonatal Fc receptor, and *CLRs* C-type lectin receptors

Target toxicity mechanisms appear when a drug interacts with its receptor. The mechanisms involved may be secondary metabolic reactions that are unwanted by the interaction of the receptor with the ADC in the presence of the target receptor in healthy tissues [2]. An example is the HER-2 receptor, which is a target for different ADCs, such as trastuzumab emtansine and trastuzumab duocarmazine, since it is overexpressed in some neoplasms (lung, ovary); however, this receptor is also found in normal corneal epithelial cells [8]. Figure 2 outlines the different mechanisms for the entry of ADC into normal cells.

## ADC-caused AEs on the ocular surface

The molecular structure, target, and current indication of ADCs are specified in Table 1. The time to onset of the ocular findings, time to the requirement to stop or reduce the dose of medication, and time to recovery of the ocular surface after cessation of therapy are specified in Table 2.

**Table 1:** Structures, targets, indications, ocular surface adverse events, and therapeutic management of antibody–drug conjugates

ADC	Structure	Target	Indication (neoplasm)	Ocular Surface AEs	Therapeutic management
AGS-16M8F	Fully human IgG2a + monomethyl auristatin F (MMAF) + maleimidocaproyl (mc) linker ^2,7,12,13^	Ectonucleotide pyrophosphatase/phosphodiesterase 3 (ENPP3) ^2,7,12,13^	Renal cell cancer (90–94% of clear cell histology and 60–69% of papillary histology) ^7^	Dry eye, blurred vision, and eye pruritis^7^	Artificial tears, steroids eye drops, or cessation of treatment^7^
AGS-16C3F				Dry eye (50%), blurred vision (44%), and keratopathy (59%)^7^	
Anetumab ravtansine (BAY 94–9343)	Fully human anti-mesothelin antibody + DM4 + cleavable disulfide linker^4,14,15^	Mesothelin^4,15^	Mesothelioma, ovarian adenocarcinomas, pancreatic adenocarcinomas, non-small cell lung cancer, gastric cancer, and triple-negative breast cancer^4,15^	Blurred vision (14–29%), keratitis (11–29%), and corneal epithelial defects (50%)^4,15^	Dose reduction, discontinuation of treatment, or use of ocular surface lubricants or topical corticosteroids^4^
Aprutumab ixadotin (BAY 1187982)	Anti-fibroblast growth factor receptor 2 (FGRF2) antibody + auristatin W derivate toxophore^17,18^	FGRF2^18^	–	Corneal calcium or lipid deposits (20%), corneal epithelial microcysts (15%), and blurred vision (5%)^18^	Carmellosa ophthalmic drops, polyacrylic acid, difluprednate, or fluorometholone drops or cessation of treatment^18^
Belantamab mafodotin (belamaf; GSK2857916)	Humanized afucosylated immunoglobulin G1 anti-B cell maturation antigen (BCMA) + MMAF + protease-resistant maleimidocaproyl cysteine linker^21^	BCMA^21^	Relapsed/refractory multiple myeloma^21^	Dry eye (34%), blurred vision (46%), foreign body sensation, and/or photophobia with keratitis and MECs (72%)^5,8,21^	Dose reductions (25%) and/or delays (47%), artificial tears, steroid eye drops, and cooling eye masks^5,8,21,22^
Coltuximab ravtansine (SAR3419, CD19-DM4)	Humanized IgG1 anti-CD19 + tubulin inhibitor maytansinoid DM4^2,23^	CD19^2^	Malignant immature B cells^2^	Blurred vision (23–41%) and corneal microcysts^2,24,25^	Dose reductions and/or delays^2,24,25^
Denintuzumab mafodotin (SGN-CD19A)	Humanized anti-CD19 + MMAF^2,24,26^	CD19^2^	B cell non-Hodgkin lymphoma (NHL)^2^	Blurred vision (35–65%), dry eyes (39–52%), and microcystic keratopathy (57–84%)^2,24,25^	Topical corticosteroids drops and modification of doses^2,25^
Depatuxizumab mafodotin (ABT-414)	Antibody ABT-806 + MMAF + non-cleavable maleimide-caproyl binding^4,27^	Amplified epidermal growth factor receptor (EGFR)^4,27^	–	Blurred vision (63%), photophobia (39%), dry eye (29%), foreign body sensation (26%), and keratitis (26%)^4,27,28^	Dexamethasone in eye drops, artificial tears, therapeutic contact lenses, or reduction and delay of dose administration^4,27,29–32^
Enfortumab Vedotin	Fully human IgG1-kappa antibody + monomethyl auristatin E (MMAE) + protease-cleavable maleimidocaproyl valine-citrulline linker (SGD-1006)^33^	Nectin-4^33^	Urothelial, breast, lung, pancreatic, and ovarian cancers^33^	Dry eye symptoms (36%), and blurred vision (14%).^33^	Artificial tears^33^
MEDI2228	Fully human BCMA antibody + pyrrolobenzodiazepine (PBD) + protease-cleavable linker^34−37^	BCMA^34−37^	Multiple myeloma^36,37^	Loss of visual acuity (54%) and dry eye (20%)^36,37^	Optimize dosage and regular comprehensive ophthalmological examination^36,37^
Mirvetuximab soravtansine (IMGN853)	Folate receptor a (FRa) binding antibody + DM4^2,4,38,42^	FRa^2,4,38,42^	Ovary, endometrium, and lung cancer with positive FRa assessed by immunohistochemistry ^2,4,42^	Blurred vision (23–50%) and keratopathy (20–32.5%)^2,38–41^	Topical ocular corticosteroids, dose calculation based on the adjusted ideal body weight, and dose reductions and/or delays^4,38,39,42^
PF-06263507 (A1-mafodotin, A1-mcMMAF, Anti-5T4 monoclonal antibody)	Humanized igg1 anti-antigen 5T4 + MMAF + non-cleavable maleimidocaproyl linker^43^	Cell-surface tumor-associated antigen 5T4^43^	–	Photophobia, dry eye, eye pain, blurred vision, conjunctivitis, increased lacrimation, keratitis, and limbal deficiency^43^	Erythromycin ointment, ophthalmic prednisolone acetate, and cessation of treatment^43^
Tisotumab vedotin	Monoclonal antibody tisotumab + MMAE + protease-cleavable valine-citrulline linker^44,46^	Tissue factor (TF)^44,46^	Cervical cancer^46^	Conjunctivitis (26%), dry eye (23%), and keratopathy (11%)^40,45,46^	Topical ocular corticosteroids or dose modification and protocol for the evaluation and monitoring of ocular events^45,46^
Trastuzumab duocarmazine (SYD985)	IgG1 monoclonal antibody anti-HER2 receptor + seco-duocarmycin-hydroxybenzamide-azaindole (seco-DUBA) + cleavable linker^47−49^	Human epidermal growth factor receptor 2 (HER2)^47^	Breast or ovarian cancer^47^	Conjunctivitis, dry eye, keratitis, and lacrimation increased^47^	Tolerability of this antibody–drug conjugate not changed with the use of prophylactic topical treatment or variations in doses or frequency of administration^47^
Trastuzumab emtansine (Ado-Trastuzumab emtansine, T-DM1)	Monoclonal antibody trastuzumab + DM1, a microtubule inhibitor^2,25,50^	HER2^4,40^	Primary human breast tumors^2,25,47^	Corneal epithelial changes, keratitis, blurred vision (4.5%), and conjunctivitis^2,25,27^	No requirement of drug discontinuation or topical treatment^50^
Vorsetuzumab mafodotin (SGN-75, CD70-MMAF)	Humanized anti-CD70 antibody + MMAF^2,51^	CD70^40^	Lymphoma, renal cell carcinoma (RCC), and glioblastoma^2^	Blurred vision (11–18%), keratitis (9%), dry eyes (27–30%), corneal epitheliopathy (15%), and corneal microcysts^2^	Artificial tears and topical corticosteroids^2^

*ADCs* antibody–drug conjugates, *AEs* adverse effects

**Table 2:** Adverse effects on the ocular surface due to antibody–drug conjugates and their temporal lapses

ADC	Time to onset of the ocular findings	Time to the requirement to stop or reduce the dose of medication	Time to recovery of the ocular surface after cessation of therapy
AGS-16M8F	Unspecified	Unspecified	Few weeks to several months^43^
AGS-16C3F			
Anetumab ravtansine (BAY 94–9343)	Unspecified	Unspecified	2–9 weeks^15^
Aprutumab ixadotin (BAY 1187982)	Unspecified	Unspecified	Unspecified
Belantamab mafodotin (belamaf; GSK2857916)	Median time to onset was 23 days (range: 1–84 days)^5,8,22^	Until the resolution of the AEs, after which it is considered to maintain a reduced dose of 1.9 mg/kg^A^	Median time to resolution was 30 days (range, 5–224 days)^8,22^
Coltuximab ravtansine (SAR3419, CD19-DM4)	Unspecified	Unspecified	1–2 weeks^2,24,25^
Denintuzumab mafodotin (SGN-CD19A)	Unspecified	Unspecified	5 weeks^25^
Depatuxizumab mafodotin (ABT-414)	Mean of 8 days^27,28^	Unspecified	Within 4 weeks to 6 months^29,30,31^
Enfortumab Vedotin	Unspecified	Unspecified	Unspecified
MEDI2228	Unspecified	Unspecified	Unspecified
Mirvetuximab soravtansine (IMGN853)	Unspecified	Unspecified	Unspecified
PF-06263507 (A1-mafodotin, A1-mcMMAF, Anti-5T4 monoclonal antibody)	Unspecified	Unspecified	Unspecified
Tisotumab vedotin	Unspecified	Unspecified	0–7 months.^46^
Trastuzumab duocarmazine (SYD985)	Unspecified	Unspecified	Unspecified
Trastuzumab emtansine (Ado-Trastuzumab emtansine, T-DM1)	Unspecified	Unspecified	Unspecified
Vorsetuzumab mafodotin (SGN-75, CD70-MMAF)	Unspecified	Unspecified	Unspecified

*ADCs* antibody–drug conjugates, *AEs* adverse effects

### AGS-16M8F and AGS-16C3F

#### Mechanism of action

AGS-16M8F binds to AGS-16 with high affinity, and this complex is internalized and trafficked to lysosomes leading to catabolism and release of active drug metabolite [12].

AGS-16C3F binds with high affinity to ENPP3. After binding, AGS-16C3F is internalized and trafficked to lysosomes, where it catabolizes and releases cysteine adducts of maleimidocaproyl monomethyl auristatin F (mcMMAF) that subsequently bind to and inhibit microtubules [13].

#### Types of AEs on the ocular surface

AGS-16M8F and AGS-16C3F are considered equivalent and have been evaluated in phase I trials [2, 7]. In the AGS-16M8F (Hyb) study (26 participants), AEs reported on the ocular surface were dry eye, blurred vision, and pruritus. These events were observed in eight patients at the three highest dose levels. Dry eye was observed in two patients at doses of 2.7 and 3.6 mg/kg, and blurred vision, dry eye, and eye pruritis were detected in six patients at a dose of 4.8 mg/kg [7].

In the AGS-16C3F (CHO) study (34 participants), the signs and symptoms were not always correlated. Dry eye and blurred vision were observed in approximately half of the patients (50% and 44% respectively), and keratopathy appeared in 59% of patients (20 patients) [7].

The severity of the AEs was dose-dependent, and at lower doses, the symptoms were better tolerated. These pre-clinical data suggest that the ocular AEs are mediated through macropinocytosis in the corneal epithelial cells, which do not express ENPP3 [7].

#### Treatment of AEs on the ocular surface

In both studies, there was a lack of information about the exact management of ocular AEs. Artificial tears and steroid eye drops were some of the treatments indicated by investigators and local ophthalmologists [7].

The keratopathy (signs and symptoms) described in both studies was reversible after drug discontinuation. In the AGS-16C3F (CHO) study, six participants discontinued the drug owing to keratopathy [7].

### Anetumab ravtansine (BAY 94–9343)

#### Mechanism of action

Anentumab ravtansine is a fully human anti-mesothelin antibody (MF-T) coupled via a reducible disulfide linker to a microtubule-targeting toxophore DM4. This combination of a linker and toxophore was selected because of its reported potential bystander effect [14].

#### Types of AEs on the ocular surface

Alterations of the ocular surface due to drug toxicity are more frequently observed in regimens with higher doses. In one study administering a dose of 6.5 mg/g every 3 weeks, among 38 patients, 29% had blurred vision and keratitis. When the dose was decreased to 2.2 mg/kg weekly, among a total of 36 patients, the prevalence of blurred vision dropped to 22%, and that of keratitis decreased to 17%; when the dose was decreased to 1.8 mg/kg weekly, among a total of 35 patients, 14% and 11% had blurred vision and keratitis, respectively [15].

In another study, corneal epithelial defects appeared in up to 50% of patients when tanezumab ravtansine was administered at a dose of 6.5 mg/kg; most cases had grades 1 and 2, and 8% had grade 3 or more according to the Common Terminology Criteria for Adverse Events (CTCAE) [4, 16].

#### Treatment of AEs on the ocular surface

Ocular AEs were managed with dose reduction, discontinuation of treatment, use of ocular surface lubricants, or topical corticosteroids. Alterations in the ocular surface subsided or a tendency toward improvement was observed in the last ocular assessment [4].

### Aprutumab ixadotin (BAY 1187982)

#### Mechanism of action

Aprutumab ixadotin binds to FGFR2 and then selectively induces cell death, through an unknown mechanism of action, in FGFR2-expressing tumor cells. FGFR2, a receptor tyrosine kinase upregulated in many tumor cell types, plays an essential role in tumor cell proliferation, differentiation and survival [17].

#### Types of AEs on the ocular surface

In a human phase I study on 20 patients with advanced, refractory solid tumors expressing FGRF2, drug-related ocular events were observed; 20% of patients had corneal calcium or lipid deposits with secondary blurred vision, 15% had corneal epithelial microcysts due to incompletely formed cells in the epithelia that cause vision hazing, and 5% had blurred vision [18].

#### Treatment of AEs on the ocular surface

In this study, topical treatment was sufficient (carmellosa ophthalmic drops, polyacrylic acid, difluprednate, or fluorometholone drops) for most patients, except one with grade 3 corneal epithelial microcysts and grade 1 blurred vision (CTCAE) [16], who had to discontinue treatment [18].

### Belantamab mafodotin (belamaf, GSK2857916)

#### Mechanism of action

B-cell maturation antigen (BCMA) or tumor necrosis factor receptor superfamily member 17 is a type III transmembrane protein that is only expressed in late memory B-cells committed to plasma cell differentiation and is present in all plasma cells, even malignant cells of multiple myeloma (MM cells). BCMA is involved in the growth and survival of long-lived plasma cells and MM cells [19, 20]. Belamaf acts through a multimodal mechanism. First, after binding to its receptor, it is rapidly internalized, and active cytotoxic drugs are released inside the cell, leading to apoptosis by inhibition of BCMA-receptor signaling and microtubule polymerization. Second, the antibody is afucosylated, which increases its binding to FcγRIIIa receptors, enhances the recruitment and activation of immune effector cells, and enhances the killing of tumor cells by antibody-dependent cellular cytotoxicity and phagocytosis. Finally, the release of markers characteristic of immunogenic cell death leads to adaptive immune response and immunological memory. The latter is shown when treatment is delayed due to ocular effects, which continue to maintain the therapeutic effect against tumor cells [11, 15].

#### Types of AEs on the ocular surface

Ocular surface involvement occurs with many conjugated antibodies with symptoms, such as eye irritation, blurred vision, and dry eye-like eye discomfort, along with signs including corneal microcyst-like epithelial changes (MECs) [8].

**Fig. 3:**
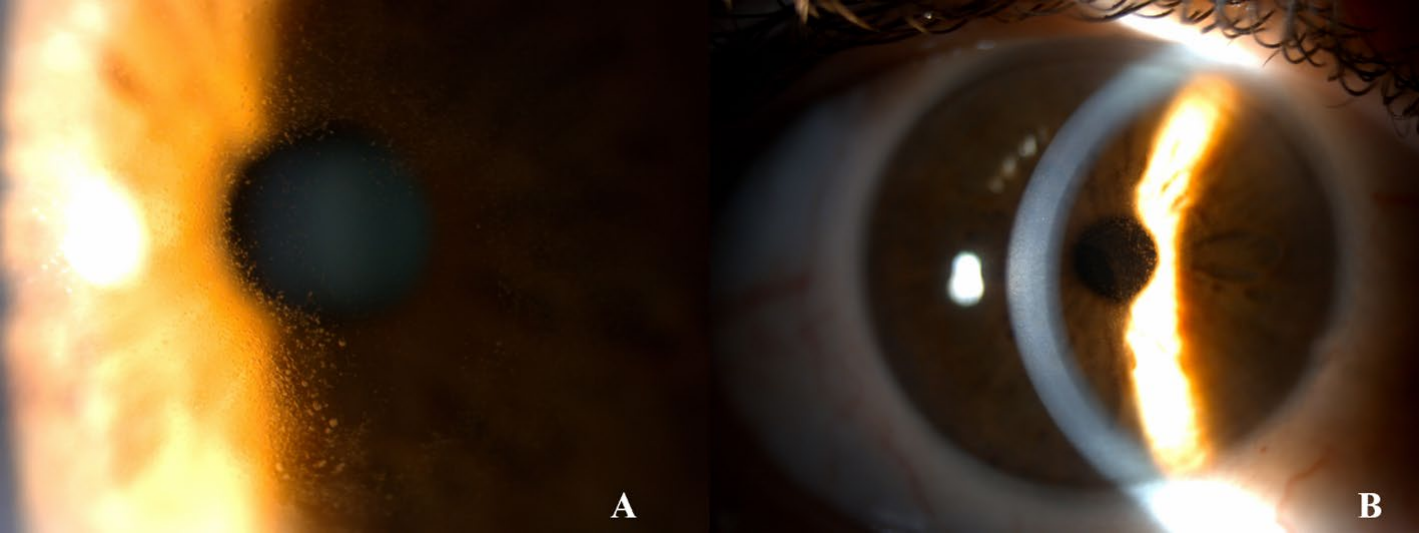
Both images, **A** and **B**, represent MECs in the central cornea in a case of keratopathy due to belantamab mafodotin at different magnifications. *MECs* microcyst-like epithelial changes

**Fig. 4:**
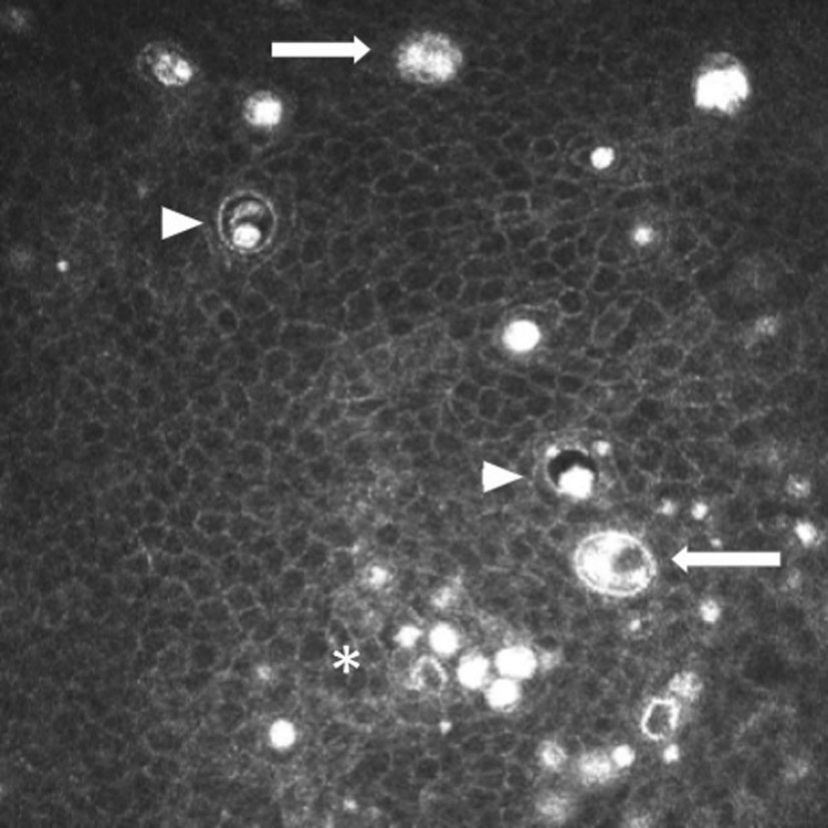
Wing cells of the corneal epithelium with intracellular hyperreflective material, even forming cell clusters as seen in the lower area (asterisk). In addition, cysts of about 3–4 cell diameters (arrowheads) and even some with several corpuscles that could be included cells (arrows)

In the DREAMM-1 study, ocular toxicity was more abundant in part-2 with a higher treatment dose than that in part-1. In part-2, the most common toxicity at the dose of 3.4 mg/kg was corneal toxicity (63% of all included cases, with 9% corresponding to grades 3–4 on the CTCAE scale) [16]. Corneal events are caused by the toxicity of the MMAF toxin, and symptoms included dry eye (34%), blurred vision (46%), foreign body sensation, and/or photophobia with keratitis and MECs (72%), all of which were reversible. MECs were observed by slit-lamp microscopy early during the treatment (69% had their first event at the dose of 4 mg/kg). Subsequently, in the DREAMM-2 study, 73% of patients had keratopathy (71% with 2.5 mg/kg belamaf versus 75% with 3.4 mg/kg belamaf), and the most common symptoms were blurred vision and dry eyes [5]. Figure 3 shows MECs, on a slit-lamp microscopy image, of a patient treated with belamaf, whereas Fig. 4 is the corneal confocal microscopy (MCC) image of MECs from the same patient.

Corneal events (dry eye and blurred vision) were found in all patients. All patients underwent corneal staining (Oxford Grading Scale). Although the Schirmer test was not included in the protocol, it was proposed that the dry eye sensation might be secondary to reduced tear production and corneal epitheliopathy [21].

In most cases, when eye damage occurs in the form of keratopathy, the patients are symptomatic. However, the absence of symptoms does not rule out the presence of corneal lesions, which increases the importance of regular ophthalmological evaluation during drug administration. For example, in the DREAMM-2 study, among all included patients who received the drug at doses of 2.5 mg/kg, 72% had MECs and 54% showed objective changes in vision; however, only 15% and 25% reported symptoms of dry eye and blurred vision, respectively [5].

Ocular AEs associated with belantamab mafodotin were more frequently detected in patients treated with higher doses, a history of dry eyes, and soluble BCMA. Although patients do not report symptoms, they may have signs of ocular toxicity; thus, it is necessary to use a slit lamp to determine the best visual acuity [6].

#### Treatment of AEs on the ocular surface

Studies found that the management of corneal events included dose reduction (25%) and/or delays (47%), artificial tears, and steroid eye drops. The dose modifications were based on changes according to a scale obtained from the best-corrected visual acuity and the degree of keratopathy (Keratopathy Visual Acuity scale) [8, 22].

Although the DREAMM-2 clinical trial ocular sub-study did not report the benefit of using topical steroids, some studies have proposed a short pulse to ameliorate symptoms [21]. However, while the use of topical corticosteroids has not shown a clear benefit in the prevention of ocular toxicity, it has been associated with a higher incidence of cataracts and glaucoma in these patients [5], Furthermore, the use of cooling eye masks is based on inducing vasoconstriction on the ocular surface during drug infusion, which theoretically should decrease the entry of ADCs at this level. However, its usefulness has not been demonstrated in various studies [5]. Lastly, close monitoring of patients by ophthalmologists has shown greater benefits in the management of AEs on the ocular surface, especially in individuals with previous ocular pathologies who have a higher risk of presenting side effects of belamaf [5].

In summary, the management of toxicity includes dosage modifications, treatment interruption or discontinuation, preservative-free artificial tears, and close ophthalmology and hematology-oncology follow-up [5].

### Coltuximab ravtansine (SAR3419, huB4-DM4)

#### Mechanism of action

Coltuximab ravtansine is an anti-CD19 monoclonal antibody conjugated to a potent cytotoxic maytansinoid, DM4, via an optimized, hindered, disulfide bond. The antibody selectively binds to the CD19 antigen present in most B cells, resulting in the internalization of the receptor-drug complex and intracellular release of DM4. DM4 is a potent inhibitor of tubulin polymerization and microtubule assembly, which ultimately induces cell apoptosis [23].

#### Types of AEs on the ocular surface

AEs on the ocular surface due to drugs vary. Blurred vision was reported in a study on 39 patients, in which after the second administration of the drug at a dose of ≥ 160 mg/m^2^, 41% of patients reported blurred vision. Similarly, at a much lower dose (55 mg/m^2^ weekly), 23% of patients developed blurred vision [2].

Corneal microcysts are also detected on ophthalmological examination and have a typical ring pattern of distribution that begins in the perilimbal area and migrates to the corneal center. Moreover, cases of accumulation of whitish corneal intraepithelial material have been reported [2]. These alterations are related to the dose administered to the patient rather than to the treatment time [24].

#### Treatment of AEs on the ocular surface

All corneal side effects were reversible by a drug delay [2, 24, 25]. Administration of the drug through guidelines with more spaced and lower doses reduces the prevalence of AEs [25].

### Denintuzumab mafodotin (SGN-CD19A)

#### Mechanism of action

Denintuzumab mafodotin is an immunoconjugate consisting of an anti-CD19 monoclonal antibody conjugated to the auristatin derivative monomethyl auristatin F (MMAF), with potential antineoplastic activity. Upon administration of denintuzumab mafodotin, the antibody moiety targets the cell surface antigen CD19, found in several B-cell-derived cancers. Upon antibody/antigen binding and internalization, the immunoconjugate releases MMAF, which binds to tubulin and inhibits its polymerization. Inhibition of tubulin polymerization may result in G2/M phase arrest and tumor cell apoptosis. This inhibits the growth of CD19-expressing tumor cells. CD19, a B-cell antigen, is overexpressed by a variety of different cancer cell types [24, 26].

#### Types of AEs on the ocular surface

After intravenous administration at doses of 0.5–6.0 mg/kg every 21 days, approximately 20% of patients had ocular AEs, among whom 59% had blurred vision, 39% had dry eyes, and 57% had microcystic keratopathy [2].

In other studies, we found that the prevalence of microcystic keratopathy, blurry vision, and dry eye were 84%, 35–65%, and 52%, respectively [24, 25].

#### Treatment of AEs on the ocular surface

The preventive administration of topical corticosteroids prior to the administration of ADC treatment reduces the incidence of severe ocular AEs (grades 3–4, CTCAE scale) [2, 16]. Once ocular alterations have been established, the use of ocular topical corticosteroids or the modification of doses can improve the clinical symptoms or result in total resolution [25].

### Depatuxizumab mafodotin (ABT-414)

#### Mechanism of action

Depatuxizumab mafodotin (ABT-414) is an ADC that binds to EGFR, which is increased in the cell surface in some tumors due to gene amplification (EGFR amplification and mutant variant 3 of EGFR amplification, formed by the deletion of exons 2 and 7). The complex is subsequently internalized, and monomethyl auristatin F (MMAF) is released by an intracellular proteolytic enzyme. MMAF inhibits microtubule function by inducing cell death [4, 27].

#### Types of AEs on the ocular surface

There are numerous different AEs on the ocular surface. In a study on 60 patients, after the administration of the drug at doses of 1.0 mg/kg every 2 weeks, a high prevalence of corneal toxicity was observed; 92% of patients were affected, with 32% being grades ≥ 3 (CTCAE scale) [4, 16].

Similar data were examined from studies where the doses ranged from 0.5–1.5 mg/kg, and toxic ocular effects were observed in 92% of patients: blurred vision (63%), photophobia (39%), dry eye (29%), foreign body sensation (26%), and keratitis (26%). Most ocular AEs were grades 1 and 2, and only 29% were grade 3; moreover, 5% of the ocular AEs were grade 4 keratitis (CTCAE scale) [16, 27, 28].

When damaged, transient amplifying cells of the cornea form small deposits or microcysts (microcystic keratopathy), which cause blurred vision, irritation, or eye pain. However, since the corneal epithelium is renewed in 21–28 days, after which epithelial alterations are self-resolved, no patient in these studies had to discontinue treatment due to ocular toxicity [27, 28].

Using MCC, it was observed that with doses of 1.5 mg/kg depatuxizumab mafodotin, multiple and diffuse hyperreflective points appeared in the corneal epithelium after 2 weeks of administration of the first dose. They persisted during treatment, and their severity increased. It has been postulated that these lesions might be early signs of epithelial cell death due to the toxicity of depatuxizumab mafodotin. After 4 weeks of treatment, round cystic structures were visualized in the epithelium and persisted throughout treatment with the fragmentation of the sub-basal nerve plexus layer. All alterations were resolved almost completely after 8 weeks of treatment [29, 30]. Moreover, some findings suggest that eye symptoms and their severity are not dose-dependent [31].

#### Treatment of AEs on the ocular surface

Artificial tears with hyaluronic acid administered three times a day from before the start of treatment and administration of corticosteroid eye drops with high doses of cytarabine have been used to prevent the formation of epithelial microcysts. This treatment is based on the fact that corticosteroids reduce cell turnover in the corneal epithelium, thereby making cells more resistant to damage from chemotherapy [27, 31]. Once ocular AEs appear, different treatment measures can be considered, such as dexamethasone in eye drops, artificial tears, therapeutic contact lenses, and reduction and delay of dose administration [32]. Corneal AEs have been demonstrated to be reversible after the discontinuation of treatment [4, 29, 30].

### Enfortumab vedotin

#### Mechanism of action

Enfortumab vedotin (EV) is an ADC whose target is Nectin-4, a transmembrane protein overexpressed in multiple cancers. Higher levels of Nectin-4 expression have been associated with disease progression and/or poor prognosis. When EV binds to Nectin-4, the ADC–Nectin-4 complex is internalized by endocytosis. The toxin of this drug is monomethyl auristatin E (MMAE), which is released via proteolytic cleavage of the linker, disrupts microtubule dynamics, and causes apoptotic cell death [33].

#### Types of AEs on the ocular surface

EV can produce ocular surface toxicity, with dry eye symptoms in 36% of patients, and blurred vision in 14% of patients, which seems to be related to dry eye [33].

#### Treatment of AEs on the ocular surface

An effective therapeutic strategy to prevent these symptoms is the use of artificial tears [33].

### MEDI2228

#### Mechanism of action

MEDI2228 carries out its action through several mechanisms. This ADC induces DNA damage responses (DDR) prior to apoptosis by phosphorylation of ATM/ATR, CHK1/2, and gH2AX in MM cells. The ATM/ATR-CHK1/2 signaling cascades activated by MEDI2228 treatment increase NKG2D ligands in MM cells and primes MM cells to natural killer (NK) cell-mediated cytotoxicity by increasing expression of MICA/B in MM cells to enhance binding and activating NK cytolytic activity. In addition, MEDI2228 stimulates STAT1- and IFN-related signaling pathways since they are activated by DDR, and both play a crucial role in innate and adaptive immunity. This leads to the overregulation of CD38, allowing greater effectiveness of drugs such as daratumumab [34, 35].

#### Types of AEs on the ocular surface

At the maximum tolerated dose (0.14 mg/kg) administered intravenously every 3 weeks, the main ocular AEs were loss of visual acuity (54%) and dry eye (20%) [36, 37].

#### Treatment of AEs on the ocular surface

Studies have shown that the best way to avoid ocular AEs is to optimize dosage and conduct regular comprehensive ophthalmological examinations [36, 37].

### Mirvetuximab soravtansine (IMGN853)

#### Mechanism of action

IMGN853 binds with high affinity and specificity to FRα, which promotes ADC internalization and intracellular release of DM4 upon antigen binding. DM4 inhibits tubulin polymerization and disrupts microtubule assembly, inducing cell cycle arrest and apoptosis [38].

#### Types of AEs on the ocular surface

The most frequently reported ocular AE in different studies is blurred vision, with a prevalence of 23–50%, which is usually reversible in all cases [2, 38, 39]. Keratopathy is also common (20–26% of cases) and can manifest as keratitis, corneal epithelial microcysts, and limbal stem cell deficiency [39].

In the phase III FORWARD I study, ocular AEs were investigated in 248 patients who received IMGN853. Among these patients, 42% (2.5% grade ≥ 3) and 32.5% (1.2% grade ≥ 3) presented blurred vision and keratopathy, respectively, which were the main reasons for interruption or reduction of the dose (CTCAE scale) [16, 40, 41].

#### Treatment of AEs on the ocular surface

Ocular AEs associated with this drug are not usually severe and are reversible. Artificial tears and periodic check-ups by a specialist ophthalmologist are used as prophylactic measures. Other useful measures include avoidance of contact lens usage, regular cleaning and use of warm compresses, and sunglasses in daylight [39]. Some authors also suggest the use of topical ocular corticosteroids [42].

The plasma drug level at the start of treatment has been demonstrated to be linked to the prevalence of AEs. Normally, the appropriate dose of the drug for each patient is calculated using the total body weight; however, one way to decrease the concentration of the drug in the blood while maintaining its effectiveness is to calculate the dose based on the adjusted ideal body weight [38]. However, when previous measures are insufficient, it is useful to modify the administered dose or suspend the drug [2, 39].

### PF-06263507 (A1-mafodotin, A1-mcMMAF, and anti-5T4 monoclonal antibody)

#### Mechanism of action

PF-06281192 recognizes a conformational epitope on the extracellular domain of human 5T4. After binding of the ADC to 5T4, the complex is internalized and catabolized in cellular lysosomes, where the active moiety of this drug, Cys-capped mc linker plus MMAF (Cys–mcMMAF, PF-06264490), is released. MMAF is an auristatin, a fully synthetic, pentapeptide inhibitor of tubulin polymerization that ultimately induces G2/mitosis cell-cycle arrest and cell death [43].

#### Types of AEs on the ocular surface

In a phase I trial, treatment-related AEs of PF-06263507 included photophobia, dry eye, eye pain, blurred vision, conjunctivitis, increased lacrimation, keratitis, and limbal deficiency. All of them, except limbal deficiency, were described in two or more patients [43].

#### Treatment of AEs on the ocular surface

Erythromycin ointment and ophthalmic prednisolone acetate, with no changes in PF-06263507 administration, were used to treat conjunctivitis, with no drug discontinuation. However, photophobia, annular keratitis, and limbal stem cell deficiency led to drug discontinuation in three patients receiving different dose regimens (4.34 or 6.5 mg/kg). Notably, the photophobia and keratitis resolved without sequelae [43].

### Tisotumab vedotin

#### Mechanism of action

Tisotumab vedotin (TV) is directed to tissue factor (TF), a transmembrane protein that initiates the coagulation cascade. TF has also been shown to play a role in tumor growth, angiogenesis, and metastasis. The antibody moiety of TV is conjugated to MMAE via a valine citrulline linker, which is proteolytically cleaved and released following the internalization of TV into cancer cells expressing TF. MMAE is a microtubule disruptor and kills actively dividing cancer cells. TV has antitumor activity on multiple tumor types and kills target cells by direct cytotoxicity, bystander cytotoxicity, antibody‐dependent cellular cytotoxicity, antibody‐dependent cellular phagocytosis, and immunogenic cell death [44].

#### Types of AEs on the ocular surface

Ocular AEs, including dry eye and corneal alterations, such as keratopathy and conjunctivitis, have been documented [40, 45]. Additionally, in the NCT03438396 study, in which a population of 101 patients was examined, ocular AEs were analyzed after administering at least one dose of tisotumab vedotin; 54% of patients had mild-to-moderate ocular AEs on the ocular surface. Among all included patients, 26% had conjunctivitis, 23% had dry eyes, and 11% had keratitis. None of these events were severe [46].

#### Treatment of AEs on the ocular surface

The use of topical ocular corticosteroids or dose modifications is very common as standard prophylactic measures. However, one of the most effective actions is the establishment of a protocol for the evaluation and monitoring of ocular events that reduce the severity of AEs [45, 46].

### Trastuzumab duocarmazine (SYD985)

#### Mechanism of action

After the interaction of trastuzumab duocarmazine with HER2, it is internalized in lysosomes, where the linker is destroyed, thereby releasing the active toxin through the alkylation of cellular DNA, which then induces cell death [47].

#### Types of AEs on the ocular surface

In a phase I dose-escalation and dose-expansion study, conjunctivitis and dry eye were two of the most common treatment-related events (both 31%). Furthermore, ocular AEs, such as keratitis and lacrimation, were reported [47].

#### Treatment of AEs on the ocular surface

The aforementioned study found that the tolerability of this ADC did not change with the use of prophylactic topical treatment or variations in dose or frequency of administration. Most patients were able to continue the study drug beyond 1 year, and most ocular events were reported as recovered or improved during the study period [47].

### Trastuzumab emtansine (ado-trastuzumab emtansine, T-DM1)

#### Mechanism of action

Trastuzumab emtansine is an ADC that incorporates the HER2-targeted antitumor properties of trastuzumab with the cytotoxic activity of the microtubule-inhibitory agent DM1 (derivative of maytansine). This ADC allows intracellular drug delivery specifically to HER2-overexpressing cells, thereby improving the therapeutic index and minimizing exposure to normal tissue [48, 49].

#### Types of AEs on the ocular surface

In a study on 20 eyes of 10 patients, low-grade corneal epithelial changes (cystoid lesions in the deep corneal epithelial cells) were found biomicroscopically by confocal microscopy. They were primarily localized in the mid-peripheral area, and no treatment-related symptoms were observed [50]. Moreover, keratitis, blurred vision (4.5%), and conjunctivitis after administration of the drug were detected [2, 25].

#### Treatment of AEs on the ocular surface

Due to the absence of symptoms during treatment, no drug discontinuation or topical treatment was required in the initial study [50].

### Vorsetuzumab mafodotin (SGN-75, CD70-MMAF)

#### Mechanism of action

Vorsetuzumab mafodotin is an ADC, whose mechanism of action is related to a humanized monoclonal antibody targeting CD70 molecule and microtubule toxin molecule MMAF. CD70 is a type II transmembrane protein, which is mainly expressed in activated T cells, B cells, NK cells, and dendritic cells. Upon binding of the ADC with CD70, the complex is internalized into the cellular lysosomes, where MMAF is released. Then, MMAF binds to tubulin and interferes with the cell cycle, causing cell death [51].

#### Types of AEs on the ocular surface

With intravenous administration at dosages of 0.3–4.5 mg/kg every 3 weeks, 57% of patients had dose-limiting ocular AEs, with blurred vision in 11%, keratitis in 9%, dry eyes in 30%, and corneal epitheliopathy in 15% of cases. By decreasing the dose to 0.3–0.6 mg/kg weekly, we found that the incidence of ocular AEs also decreased by up to 36%; the incidence of blurred vision decreased by 18%, and that of dry eyes was reduced by 27% [2].

Additionally, cases of corneal microcysts have been described, and their pattern of appearance consists of lesions in the corneal periphery that advance toward the central region, with the appearance of secondary refractive alterations [2].

#### Treatment of AEs on the ocular surface

The duration and severity of ocular AEs were reduced by treatment with artificial tears and topical corticosteroids [2].

## Conclusion

ADCs are a promising therapy used in oncology as the last line of treatment, focusing on maximizing efficacy with minimal associated AEs. However, they are associated with significant ocular toxicity that could result in mild discomfort and significant visual loss, which may lead to the cessation of the treatment. Despite the significant impact on the line of treatment caused by the loss of patient well-being as a result of AEs on the ocular surface, there are not enough studies that measure the impact they produce on the patient's quality of life in a standardized manner.

Currently, the mechanisms by which these alterations appear at the ocular surface are unclear; however, knowledge of the pathophysiology of ADCs would help us improve their design, thereby allowing the largest number of patients with indications to benefit from them. It is known that AEs are influenced by the dose of treatment and the frequency of administration in most cases, where the higher the dose or frequency, the greater the AEs. The appropriate dose and frequency of administration vary depending on the drug and the patient in question, and so it is a challenge to determine the most appropriate regimen in each case. A detailed analysis of the AEs caused in different doses with large-scale studies could be very useful in decision-making.

The statistical analysis of data related to AEs on the ocular surface due to the use of ADCs is difficult because of the scarcity of published literature, the small number of patients in each study, and the great variability of interpersonal conditions within the same study and different studies. Furthermore, little information is available regarding the diagnostic methods used in the ophthalmological follow-up of patients and the specific findings identified on the ocular surface. These factors make it difficult to perform powerful analyses.

The chemical macrostructure of ADC seems to influence its behavior at the plasma level and its interaction with cells of different tissues. Different types of linkers used in the binding of drug components can facilitate the release of cytotoxins into the extracellular medium, resulting in unwanted toxicity. Similarly, the higher the affinity of the monoclonal antibodies to the target receptor, the lesser the damage to healthy tissues, thus avoiding unwanted effects and vice versa. Therefore, it is very important to consider AEs when devising new ADCs, and it is essential to study, monitor, and analyze the side effects of drugs marketed to date in detail.

Alterations of the ocular surface are among the most common AEs in the literature and are reversible after cessation or delay of treatment in general. The preventive and therapeutic measures used vary, but those with greater effectiveness are associated with hydration through artificial tears and close monitoring by an ophthalmologist, which allow the identification of important alterations that are not always accompanied by obvious symptoms. Topical ocular corticosteroids are widely used in different studies, most of which agree that their use does not lead to significant symptom improvement. Long-term topical ocular corticosteroid use may be associated with important side effects.

In summary, because of the limited findings in this therapeutic field, it is important to conduct multidisciplinary monitoring with specialists in oncology, hematology, and ophthalmology to allow the diagnosis and treatment of these symptoms and signs.

## Literature search

Using the PubMed and Scopus platform as databases, we performed a bibliographic search using descriptors, such as ‘Inmunoconjugates’, ‘Antibody–Drug Conjugate’, ‘adverse effect’, ‘side effect’, ‘adverse event’, ‘toxicity’, ‘ocular’, ‘ophthalmolog*’, or ‘eye’. As a result of the newness of antibody–drug conjugates, we selected reviews published since 2015, with some exceptions in terms of the search for mechanisms of action of drugs. After preliminary analyses of the results, reviews related to the study topic were selected.

With the information obtained, we subsequently conducted bibliographic searches that focused on the selection of specific ADCs associated with AEs on the ocular surface described by the scientific community in relation to this type of therapy using descriptors, such as ‘Blurred vision’, ‘Keratitis’, ‘Dry eye’, ‘Corneal microcysts’, ‘Corneal deposit’, ‘Corneal inclusion’, ‘Conjunctivitis’, ‘keratoconjunctivitis’, ‘Corneal epithelial defect’ or ‘Corneal epithelial damage’, and ‘Inmunoconjugates’ or ‘Antibody–Drug Conjugate’.

## Data Availability

Not applicable.
